# Aneuploidy in spermatids of Robertsonian (Rb) chromosome heterozygous mice

**DOI:** 10.1007/s10577-014-9443-7

**Published:** 2014-11-11

**Authors:** Catalina Manieu, Marisel González, Julio López-Fenner, Jesús Page, Eliana Ayarza, Raúl Fernández-Donoso, Soledad Berríos

**Affiliations:** 1 Programa Genética Humana, ICBM, Facultad de Medicina, Universidad de Chile, Santiago, Chile; 2 Departamento de Ingeniería Matemática, Universidad de La Frontera, Temuco, Chile; 3 Departamento de Biología Celular, Universidad Autónoma de Madrid, Madrid, Spain

**Keywords:** Rb heterozygotes, Trivalent segregation, Spermatid aneuploidy, *Mus musculus domesticus*

## Abstract

Rb translocations are chromosomal rearrangements frequently found in natural populations of the house mouse *Mus musculus domesticus*. The standard diploid karyotype of the house mouse consisting of 40 telocentric chromosomes may be reduced by the emergence of metacentric Rb chromosomes. Multiple simple Rb heterozygotes form trivalents exhibiting higher anaphase nondisjunction frequency and consequently higher number of unbalanced gametes than in normal males. This work will attempt to establish whether frequencies of aneuploidy observed in heterozygote spermatids of the house mouse *M. musculus domesticus* show differences in chromosomes derived from different trivalents. Towards this goal, the number and distribution frequency of aneuploidy was assessed via FISH staining of specific chromosomes of spermatids derived from 2n = 32 individuals. Our results showed that for a given set of target chromosomes, 90 % of the gametes were balanced, resulting from alternate segregation, and that there were no differences (approx. 10 %) in aneuploidy frequencies in chromosomes derived from different trivalents. These observations suggest that segregation effectiveness does not depend on the type of chromosomes involved in trivalents. As a consequence of the trivalent’s configuration, joint segregation of the telocentric chromosomes occurs thus favoring their appearance together in early spermatids. Our data suggest that Rb chromosomes and their telocentric homologs are subject to architectural constraints placing them close to each other. This proximity may ultimately facilitate fusion between them, hence contributing to a prevalence of Rb metacentric chromosomes.

## Introduction

Rb translocations involve double-strand DNA breaks at the centromere level in two telocentric (acrocentric) chromosomes followed by repair (fusion) ligating the respective long arms creating a metacentric Rb chromosome. The short arms (p) of the original telocentric chromosomes, including the proximal telomeres, part of the satellite DNA, and frequently one centromere, are lost (Comings and Avelino 1972; Nanda et al. 1995; Garagna et al. 2001a, 2002).

Rb translocations are frequently present in natural populations of the house mouse *Mus musculus domesticus*. As a consequence, the standard diploid karyotype of the house mouse consisting of 40 telocentric chromosomes may be reduced by the emergence of metacentric Rb chromosomes. This natural process has produced more than 40 different chromosomal races, ranging from 2n = 40 to 2n = 22 (Gropp et al. 1982; Nachman and Searle 1995; Capanna and Redi 1995; Piálek et al. 2005). About 100 Rb chromosomes with different combinations of arms have been characterized (Redi and Capanna 1988), many of which emerged and spread extremely rapidly among populations carrying the standard karyotype (Britton-Davidian et al. 2000).

Two distinct types of Rb heterozygotes have been recognized (Redi and Capanna 1988; Searle 1993): (1) complex heterozygotes which carry two or more metacentric chromosomes with common chromosome arms that consequently form rings or chains of more than three chromosomes at meiosis I and (2) simple or multiple simple heterozygotes which carry one or more metacentric chromosomes and homologous telocentric forming trivalents during meiosis I (Wallace et al. 2002).

In a trivalent, a Rb metacentric chromosome is synapsed with the long arms of two homologous telocentric chromosomes. The short arms of telocentric chromosomes exhibit total or partial synapse or complete asynapsis (Manterola et al. 2009). The asynaptic axes show ectopic joints either with other trivalents or with the X chromosome of the XY bivalent which could affect their subsequent segregation (Manterola et al. 2009; Berrios et al. 2014). The asynapsis between heterologous regions of these telocentric chromosomes may trigger meiotic silencing of unsynapsed chromatin (MSUC) which may lead to apoptosis (Mahadevaiah et al. 2008; Manterola et al. 2009). Consequently, the asynapsis of multivalents and/or interactions between XY chromosomes and trivalents may also affect survival of germ cells by affecting their normal gene expression (Everett et al. 1996; Burgoyne et al. 2009; Garagna et al. 2001b; Homolka et al. 2007; Mahadevaiah et al. 2008). Based on these observations, it has been proposed that Rb hybrids succeeding in completing meiosis producing viable gametes are strongly determined by the complete synapses of their trivalents (Grao et al. 1989; Redi et al. 1990, 2001).

**Fig. 1:**
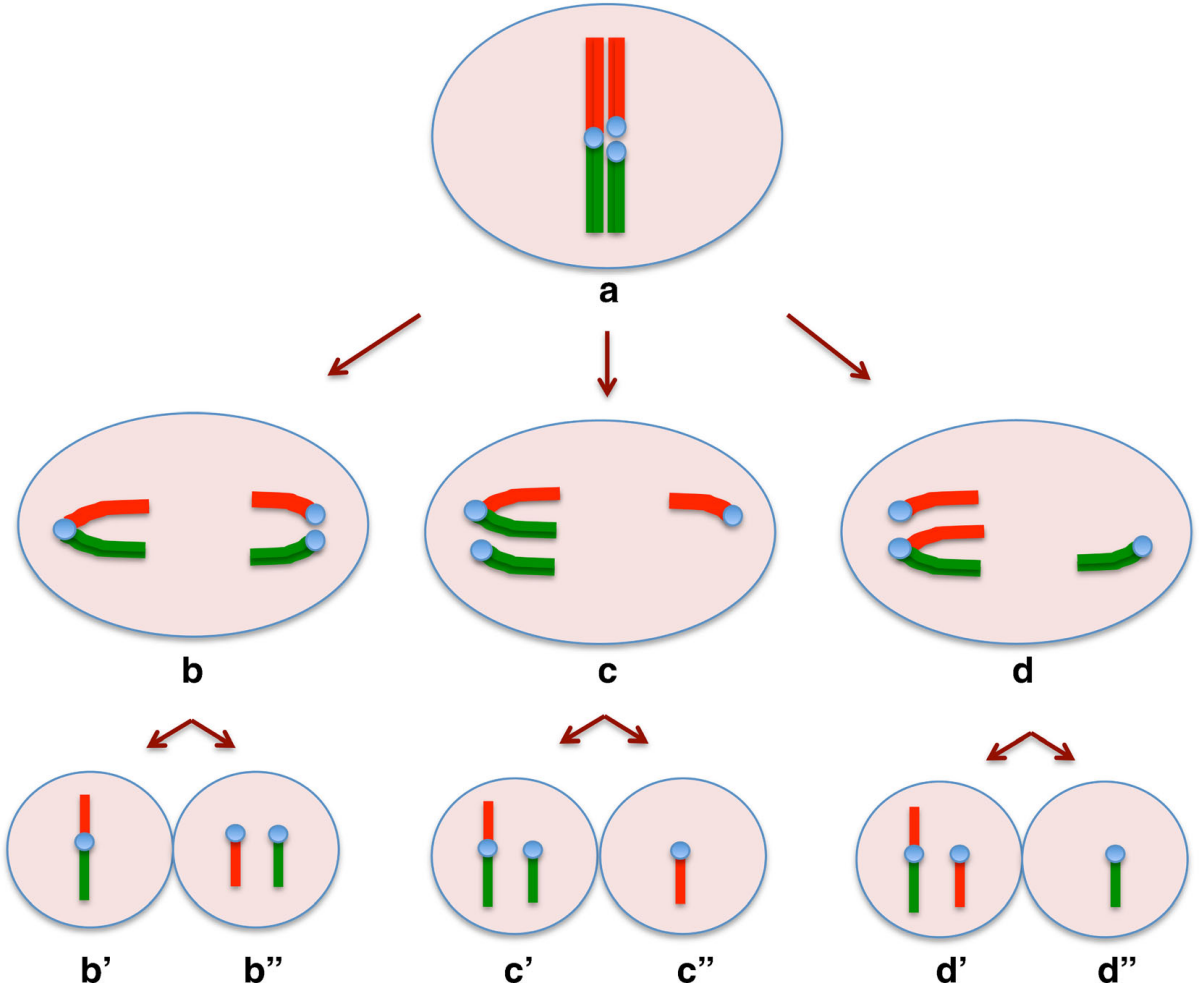
Diagram of trivalent, options of chromosome segregation and resulting gametes. (*a*) Configuration of the three chromosomes in a trivalent at meiosis of a heterozygous 2n = 32. According to FISH and the specific DNA probes, one arm of the metacentric chromosome is in *red* (Cy2) and the other one in *green* (FITC) as the respective telocentric homologous chromosomes. Only one trivalent is depicted. (*b*) alternate segregation; (*c*) and (*d*) adjacent segregations; (*b*′) balanced and (*b*″) normal gametes; (*c*′), (*c*″), (*d*′), and (*d*″) aneuploid gametes

Although Rb heterozygotes have an increased loss of spermatocytes at prophase I, a large number of them reach the meiotic divisions, where finally trivalent chromosomes should segregate (Garagna et al. 2001b). The chromosomes of each trivalent must move together into alignment at metaphase and must separate from each other at anaphase I. At this time, abnormal segregation (nondisjunction) of Rb heterozygotes is enhanced by their susceptibility to orientate incorrectly (Eichenlaub-Ritter 1994). Trivalent segregation falls into one of three modes: alternate, adjacent, or 3:0 segregation, producing eight different gametes (Anton et al. 2010). Only gametes resulting by alternate segregation exhibit normal or balanced karyotypes. Gametes produced by other segregation modes have unbalanced karyotypes, being nullisomic or disomic for one or more chromosomes (Fig. 1).

The metaphase checkpoint activates cellular mechanisms that interrupt the spermatocyte’s meiotic progress when the spindle assembly or alignment of chromosomes at the equatorial plane occurs incorrectly. During this process, several corrective action events are triggered. However, if correction is not effective and the errors persist, the spermatocyte’s fate can be either apoptosis or the completion of the meiotic process, thereby transferring errors to spermatids (Handel et al. 1999; Eaker et al. 2001; Merico et al. 2008; Gillies et al. 2013).

Multiple heterozygotes with higher number of trivalents are known to exhibit lower germ cell counts and higher abnormal segregation frequencies during anaphase I (Wallace et al. 2002). However, it remains unclear whether abnormal segregation depends upon the chromosomes involved in a trivalent and whether the frequency of aneuploid spermatids vary for each trivalent from a multiple heterozygote. It has been reported elsewhere that in heterozygote mice carriers of two trivalents, adjacent disjunction derived from one trivalent is very different from the other disjunction (Winking et al. 2000). While a different performance of the centromeric regions has been suspected of being responsible for these differences, to our knowledge no clear explanation of this behavior has been reported so far.

In this work, the impact of specific trivalents upon meiotic chromosomal segregation of heterozygotes carrying multiple Rb chromosomes was scrutinized. This was accomplished by identifying the presence or absence of chromosomes derived from specific trivalents and obtaining corresponding spermatid aneuploidy frequencies (Fig. 1).

## Materials and methods

Four male mice were used for this study. Two were heterozygote 2n = 32. The other two were homozygotes: one 2n = 40 and the other one 2n = 24. Heterozygotes were generated by crossing two females of the laboratory strain CD1 2n = 40, with all their chromosomes telocentric, with two males of the Milano II race 2n = 24 with eight pairs of homozygote Rb metacentric chromosomes.

The Rb chromosomes are the following: Rb 2.12, 3.4, 5.15, 6.7, 8.11, 9.14, 10.13, 16.17. Numbers are according to the 2n = 40 standard karyotype. Mice were maintained at 22 °C with a light/dark cycle of 12/12 h and fed ad libitum. Procedures involving the use of the mice were reviewed and approved by the Ethics Review Committee of the School of Medicine, Universidad de Chile (No. CBA #0441) and by the Ethics Review Committee of the Chilean National Science Foundation FONDECYT-CONICYT. Care and handling of laboratory animals followed all institutional and national guidelines (protocol CBA #0441 FMUCH).

### Germ cells suspensions

Testes were extracted and the albuginea removed leaving the seminiferous tubules exposed. Seminiferous tubules were placed in cold phosphate-buffered saline (PBS) and minced with tweezers to release germ cells. The resulting homogenate was let to stand for 10 min at room temperature, the supernatant collected, and germ cells collected by centrifugation at 70×*g*. Pellets enriched in germ cells were resuspended in cold PBS and fixed in cold 3:1 (*v*/*v*) 100 % (*v*/*v*) methanol/100 % (*v*/*v*) acetic acid for 20 min at 4 °C. After fixation, germ cells were collected by centrifugation at 70×*g* and resuspended in fresh fixative as above. Fifty microliters of aliquots of the germ cell suspension were placed onto clean microscope slides and air-dried at room temperature before use.

### In situ hybridization

We analyzed the aneuploidy of round spermatids from two heterozygote Rb mice 2n = 32, both with the same set of eight single Rb metacentric chromosomes. 2n = 32 Rb heterozygotes were compared with the parental homozygotes. Chromosomes 5, 9, 14, 15, 16, or 17 were identified by in situ hybridization using commercially available probes (MetaSystems, Germany). These chromosomes are Rb metacentric in the following pairs: Rb 5.15, 9.14, and 16.17. Chromosomes 15 and 16 are carriers of nucleolar organizing region (NOR), while chromosomes 5, 9, 14, and 17 have no NOR (Cazaux et al. 2011; Britton-Davidian et al. 2012).

Slides containing germ cells prepared as described above were treated for 5 min with PBS; dehydrated in a series of 70, 80, 90, and 100 % ethanol for 2 min each; and air-dried at room temperature. DNA probes specific for two chromosomes were added to germ cells, mounted with coverslips, and denatured together at 75 °C for 2 min. Following denaturation, slides were incubated in a humid chamber at 41 °C for 16 h. After incubation, coverslips were removed, and slides rinsed with 0.4× saline sodium citrate (SSC) buffer at 72 °C for 2 min; 2× SSC, 0.05 % (*v*/*v*) Tween 20 (Sigma-Aldrich) at room temperature for 30 s. Finally, cells were rinsed twice in PBS for 5 min each. Nuclei were stained with 4′,6-diamino-2-phenylindole (DAPI) (Calbiochem) and coverslips mounted with Vectashield (Vector Laboratories). Images were acquired using a Nikon (Tokyo, Japan) Optiphot or an Olympus BX61 epifluorescent microscopes equipped with Nikon PL APO 100X, 1.30 NA objective lenses. Corresponding fluorescent signals were detected using the following barrier filters Chroma 49002 ET-GFP (FITC/Cy2), 49010 ET-R&B Phycoerythrin/mOrange/Mko y Hoetch UV-2A. Images were acquired using a DS-L1 Nikon camera control unit. All images were minimally processed for contrast using Adobe Photoshop CS5.1 software.

### Data quantification

Chromosome segregation was assessed using three pairs of probes. For each heterozygote, chromosome segregation of three trivalents was assessed separately as follows: (a) 5(5.15)15, (b) 9(9.14)14, (c) 16(16.17)17.

One hundred spermatids for each chromosome pair, namely 5,15; 9,14; 16,17, were studied for each individual, two homozygotes and two heterozygotes, yielding a universe of about 1,200 spermatids. The criterion used for signal discrimination and chromosome differentiation was as described by Moradkhani et al. (2006a, b) and Vozdova et al. (2008). Discrimination between metacentric and telocentric chromosomes inside the spermatid’s nuclei required that fluorescent signals be separated by at least a distance equivalent to the size of the domain recognized by one of the specific probes. Segregation pattern analysis was performed using a two-tailed Fischer’s test, with a *p* value <0.05 for establishing statistically significant differences.

## Results

### Chromosome identification in spermatids from homozygotes 2n = 40 and 2n = 24 of *M. musculus domesticus*

Identifiable cells obtained from seminiferous tubules were as follows: spermatogonia, spermatocytes in prophase I or in metaphases and anaphases II, spermatids, spermatozoa, and Sertoli cells. Chromosome observations were performed in early spermatids where the nucleus was still spherical and chromatin was lax, heterogeneous and presenting a large central chromocenter intensely stained with DAPI. These spermatids were chosen because larger nuclei favored recognizing whether chromosomes identified by fluorescent painting were together or apart. For each nucleus examined, only one pair of chromosomes was identified each time: 5 and 15, 9 and 14, or 16 and 17.

**Fig. 2:**
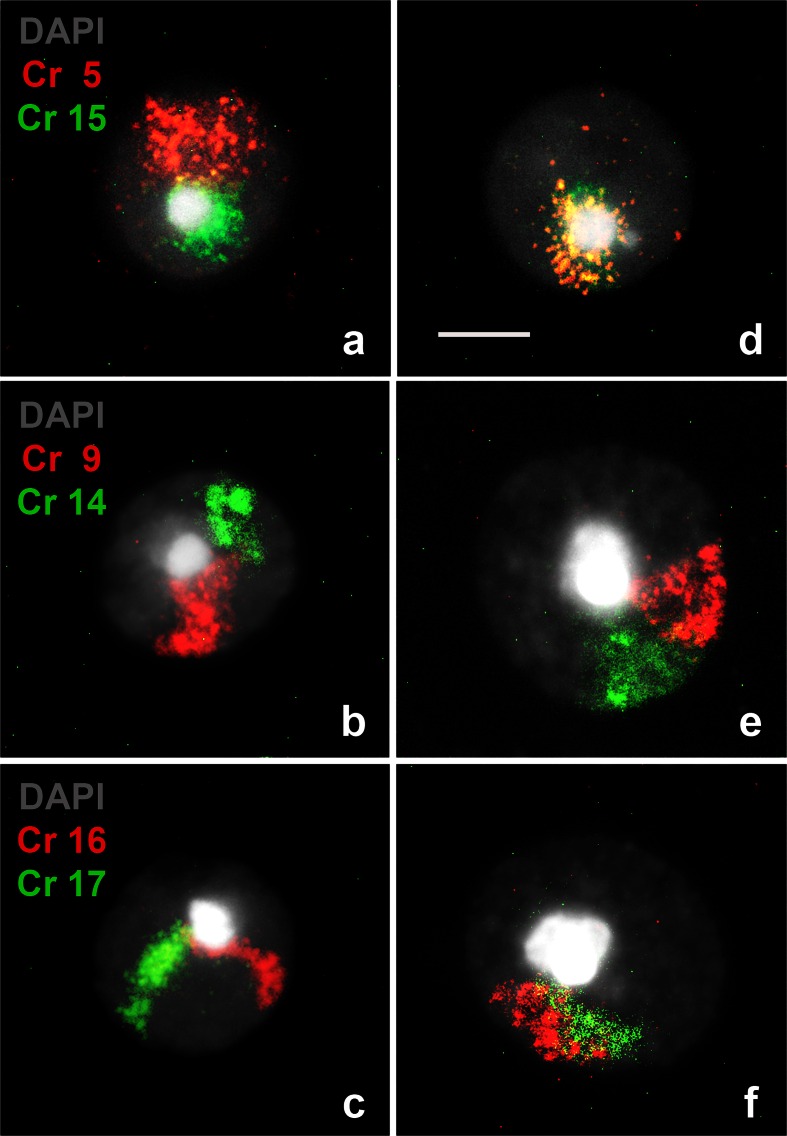
Chromosome in spermatids from homozygotes 2n = 40 and 2n = 24 of *M. musculus domesticus*. Nuclei of spermatids of individuals 2n = 40 (**a**–**c**) and 2n = 24 (**d**–**f**) are shown. In *gray* the homogeneous chromatin and *white* the central chromocenter, both stained with DAPI. Chromosomes were stained with FISH: 5, 9, and 16 are observed in *red*; 15, 14, and 17 in *green*. In **a**, chromosomes 5 and 15; in **b**, chromosomes 9 and 14; and in **c**, chromosomes 16 and 17. They all are connected only through the chromocenter. In contrast, in **d**–**f**, both signals corresponding to the arms of the respective Rb metacentric chromosomes are also connected through the chromocenter but now they appear side by side. *Bar* = 5 μm

Nuclei from homozygote individuals showed a red fluorescent signal corresponding to chromosome 5, 9, or 16 and a green fluorescent signal, corresponding to chromosome 15, 14, or 17 (Fig. 2). In spermatids of 2n = 40 individuals, we observed that chromosomes from each pair appeared joined only through their pericentromeric heterochromatin (Fig. 2a–c; Fig. 6(*b′*)), while in most spermatids of 2n = 24 individuals, both fluorescent probes colocalized or were found together side by side towards a single chromocenter (Fig. 2d–f; Fig. 6(*a′*)). From the 600 homozygous spermatids analyzed, only one from a 2n = 40 individual presented an extra red fluorescent signal corresponding to chromosome 9.

### Chromosome identification in spermatids from heterozygotes 2n = 32 of *M. musculus domesticus*

One hundred spermatids from each of two 2n = 32 heterozygotes were evaluated for each of the following chromosomal pairs: 5,15; 9,14 and 16,17.

**Fig. 3:**
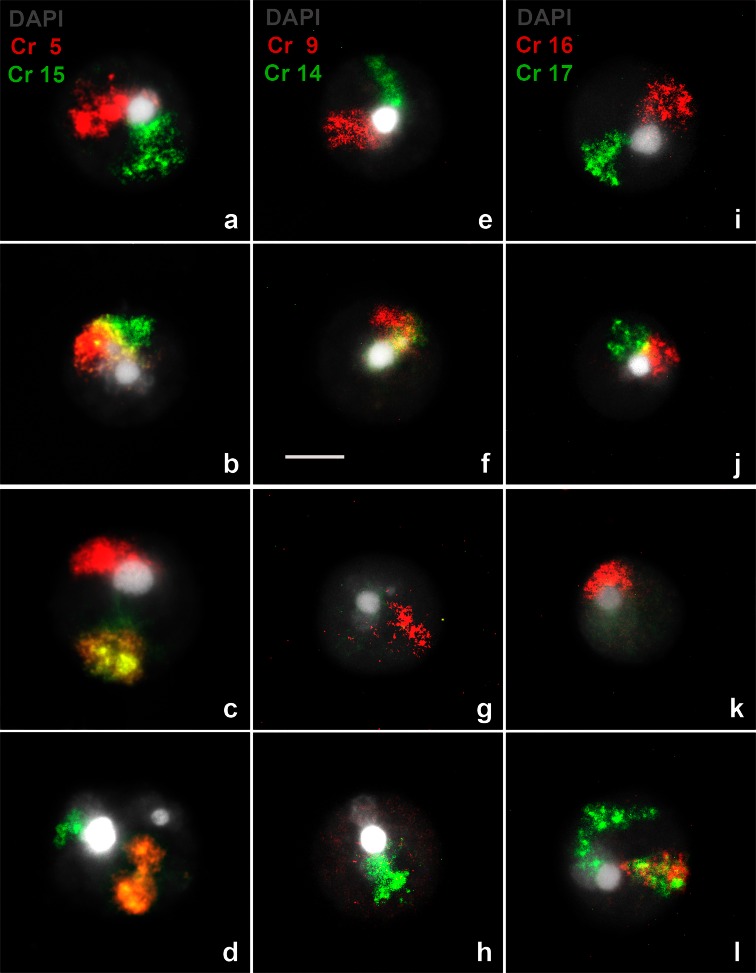
Normal and aneuploid spermatids of Rb heterozygotes 2n = 32. Chromosomes 5, 9, and 16 in *red* and chromosomes 15, 14, and 17 in *green* were stained by FISH, while the nuclei were counterstained with DAPI. Normal or balanced spermatids with respect to chromosomes 5 and 15 (**a**, **b**), 9 and 14 (**e**, **f**), and 16 and 17 (**i**, **j**). In **a**, **e**, **i**, chromosomes or chromosome arms are connected together only by their heterochromatin to the chromocenter, whereas in **b**, **f**, **j**, chromosomes appear adjacent to each other and also connected to the chromocenter. Aneuploid spermatids: disomy of chromosome 5 (**c**), disomy of chromosome 15 (**d**); nullisomy of chromosomes 14 and 9, (**g**, **h**, respectively); nullisomy of chromosome 17 (**k**) and disomy of chromosome 17 (**l**). *Bar* = 5 μm

**Fig. 4:**
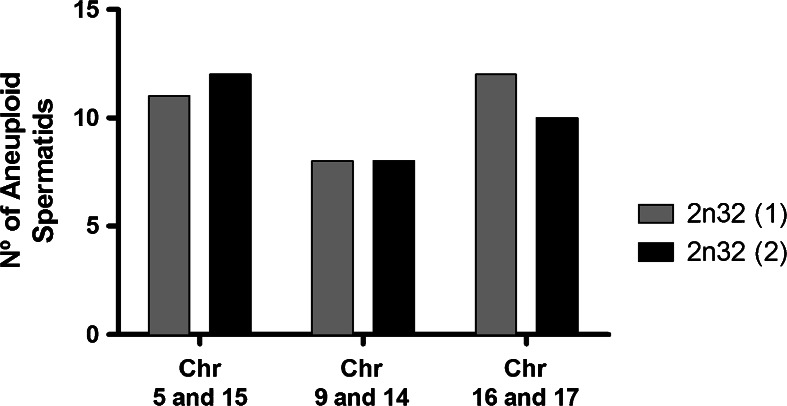
Frequency of aneuploid spermatids in 2n = 32 heterozygotes. By FISH and specific DNA probes, the spermatid nuclei for three chromosomal pairs were examined. The Fischer test shows no significant differences between the frequency of aneuploidy for the indicated chromosomal pairs. (*p value >0.05)

Chromosomal probes 5 and 15 showed that 88.5 % of spermatids resulted from alternate segregation (Fig. 3a, b), while the remaining 11.5 % resulted from adjacent meiotic segregation (Fig. 3c, d; Table 1). When evaluating spermatids with probes 9 and 14, we found that 92 % were product of alternate segregation (Fig. 3e, f), while only 8 % resulted from adjacent segregation (Fig. 3g, h; Table 2). In the case of chromosomes 16 and 17, it was observed that 90 % of the spermatids were product of alternate segregation (Fig. 3i, j), while the remaining 10 % resulted from adjacent segregation (Fig. 3k, l; Table 3). Comparing the frequencies of aneuploid spermatids from different heterozygote individuals, we found no significant differences for any of the chromosome pairs studied (Fig. 4).

**Table 1:** Normal or aneuploid spermatids of 2n = 32 heterozygotes. Chromosomes 5 and 15

Spermatids	Individual 1	Individual 2
Normals	89	88
Aneuploids	11	12
Chromosomal aneuploidies	Individual 1	Individual 2
Disomy cr. 5	4	5
Disomy cr. 15	5	3
Disomy cr. 5 and 15	1	2
Nullisomy cr. 5	0	1
Nullisomy cr. 15	1	1
Nullisomy cr. 15 and 5	0	0

**Table 2:** Normal or aneuploid spermatids of 2n = 32 heterozygotes. Chromosomes 9 and 14

Spermatids	Individual 1	Individual 2
Normals	92	92
Aneuploids	8	8
Chromosomal aneuploidies	Individual 1	Individual 2
Disomy cr. 9	1	4
Disomy cr. 14	1	2
Disomy cr. 9 and 14	0	1
Nullisomy cr. 9	3	0
Nullisomy cr. 14	2	1
Nullisomy cr. 9 and 14	1	0

**Table 3:** Normal or aneuploid spermatids of 2n = 32 heterozygotes. Chromosomes 16 and 17

Spermatids	Individual 1	Individual 2
Normals	88	90
Aneuploids	12	10
Chromosomal aneuploidies	Individual 1	Individual 2
Disomy cr. 16	0	3
Disomy cr. 17	7	4
Disomy cr. 16 and 17	0	1
Nullisomy cr. 16	1	0
Nullisomy cr. 17	2	1
Nullisomy cr. 16 and 17	2	1

Among the observed 11.5 % aneuploid spermatids labeled with probes 5 and 15 (23 spermatids), nine were carrying an extra chromosome 5 (Fig. 3c), eight had an extra chromosome 15 (Fig. 3d), three had two chromosomes 5 and one chromosome 15 and, only one was nullisomic for chromosome 5, whereas two were nullisomics for chromosome 15. Nullisomic spermatids for both chromosomes were not found (Table 1).

Among the 8 % aneuploid spermatids tagged with probes 9 and 14 (i.e., 16 spermatids), five carried a disomy for chromosome 9, three showed an extra chromosome 14, one exhibited a disomy for chromosomes 9 and 14, three had nullisomy for chromosome 9 (Fig. 3g), and three were nullisomics for chromosome 14 (Fig. 3h). Only one was found to be nullisomic for both chromosomes (Table 2).

Among the 10 % aneuploid spermatids tagged with probes 16 and 17 (22 spermatids), 3 of them carried an additional chromosome 16, 11 carried an extra chromosome 17 (Fig. 3l), and 1 a disomy for both chromosomes. Only one was nullisomic for chromosome 16, three were nullisomics for chromosome 17 (Fig. 3k), and three were nullisomics for both chromosomes 16 and 17 (Table 3).

Frequency differences between aneuploid spermatids of homozygotes and heterozygotes for all chromosomes studied were statistically significant in all cases (*p* < 0.001). In contrast, frequency differences between aneuploid spermatids of heterozygotes for the different chromosomic pairs were not statistically significant (*p* > 0.05).

### Arrangement of chromosomes or chromosome arms in spermatid nuclei from homozygote or heterozygote males

In spermatids of different chromosomal constitution, stained by FISH and DAPI, the relative nuclear position of specific chromosomal pairs and their pericentromeric heterochromatin was studied.

In all the spermatids, the chromosome pairs or chromosomal arms were connected through heterochromatin to a big chromocenter localized at the nucleus center.

Approximately 70 % of spermatids from 2n = 40 individuals exhibited a configuration in which the chromosome pairs (5,15; 9,14; and 16,17) were found to be connected only through the chromocenter, while the remaining 30 % were additionally found to be lying adjacent to each other (Fig. 2a–c).

A different story shows up when performing the same analysis, but now on 2n = 24 individuals: it was observed that for approximately 95 % of all spermatids, both chromosomal arms were found lying adjacent and only 5 % were found separated (Fig. 2d–f).

**Fig. 5:**
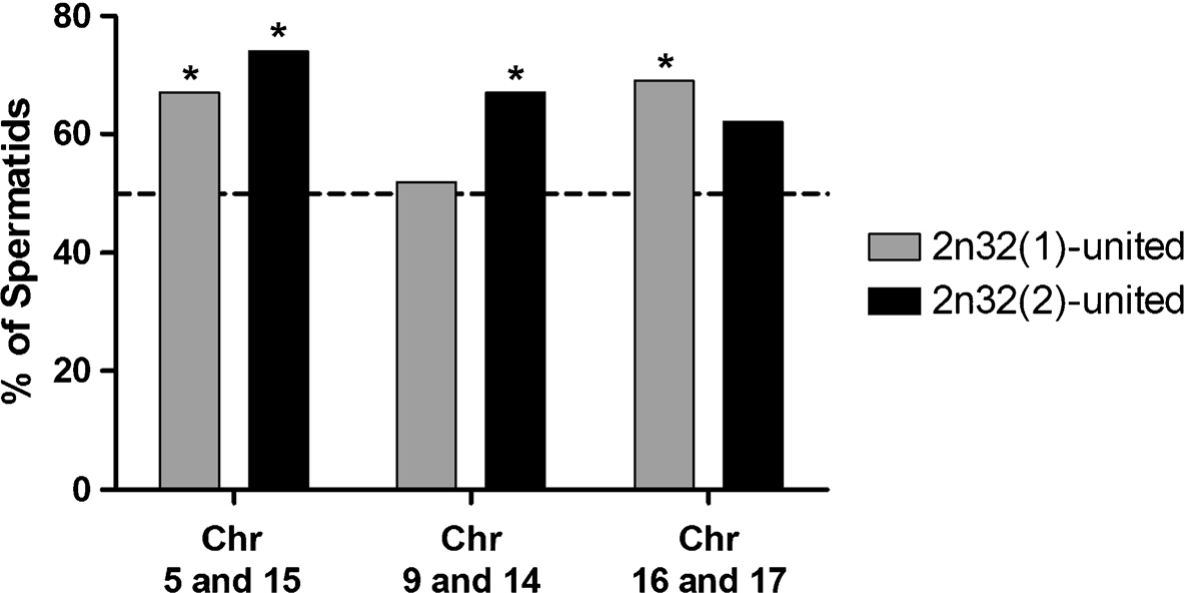
Arrangement of chromosomes or chromosome arms in the spermatid nuclei from 2n = 32 heterozygotes stained with FISH. The pairs of chromosomes or chromosomal arms appear side by side in four out of six individuals (*p < 0.05)

The same analysis performed in normal or balanced spermatids from 2n = 32 heterozygotes proved adjacent localization for all three chromosomal probes to be the most frequent chromosomal arrangement (Table 4). In four of six cases studied, differences in favor of adjacent fluorescent signals (Fig. 3b, f, j) versus separated ones (Fig 3a, e, i) were statistically significant (*p* < 0.05) (Table 4, Fig. 5).

**Table 4:** Frequencies of observed chromosome distributions within the nucleus in spermatids of two different 2n = 32 individuals

	2n32 (1)			2n32 (2)		
Chromosomes	United (%)	Not united (%)	*p* value	United (%)	Not united (%)	*p* value*
5 and 15	67	33	0.0214*	74	26	0.0008*
9 and 14	52	48	0.8876	67	33	0.0214*
16 and 17	69	31	0.0093*	62	38	0.1169

*p* value between observed and expected frequencies. For all cases the expected frequency was 0.5

*p < 0.05 (significant)

## Discussion

Rb translocation is the most common chromosomal rearrangement in mammals (King 1993) and represents the type of chromosomal change that most effectively contributes to differentiation or speciation of natural (wild) populations (Garagna et al. 2001a). In wild populations of *M. musculus domesticus*, it is frequent to find the occurrence of centric fusions between two telocentric chromosomes, giving rise to Rb metacentric chromosomes (Redi and Capanna 1988). Rb heterozygote males generate trivalents and a peculiar nuclear architecture on their meiotic prophase cells (Wallace et al. 2002; Berrios et al. 2014). In trivalents, asynapsis or delay in synapse during prophase may occur (Mahadevaiah et al. 2008; Manterola et al. 2009) and difficulties in chromosome segregation during meiotic divisions as well (Searle 1993; Everett et al. 1996; Eaker et al. 2001). A trivalent can be subject to either alternate segregation, adjacent segregation, or 3:0 segregation (Anton et al. 2010). In human male carriers of Rb translocations, alternate segregation has been described as being the most prevalent. Normal or balanced spermatozoa are present between about 77 and 93 %, while aneuploid spermatozoa, which result from adjacent segregation, has been reported as to be present between about 7 and 23 % (Anton et al. 2004; Ogur et al. 2006; Bernicot et al. 2012; Pylyp et al. 2013).

Similar values were observed with spermatids from two 2n = 32 heterozygotes of *M. musculus domesticus* studied: 88 to 92 % were found to be normal or balanced spermatids, whereas 8 to 12 % were aneuploid. No difference in the frequencies of aneuploid spermatids was found between individuals. This is most likely due to their similar genetic and chromosomal lineage. It is important to note that our estimations only considered aneuploidy resulting from segregation of chromosomes involved in only one trivalent. We do not know what would happen in the case of simultaneous chromosome segregation of the eight trivalent present on 2n = 32 heterozygotes. To estimate the total frequency of aneuploidy for multiple Rb heterozygous, it has been proposed by others to multiply aneuploidy frequency of one Rb chromosome by the total number of Rb chromosomes (Eaker et al. 2001). We do not support estimating the total aneuploidy in this fashion because segregation of one trivalent could be independent from segregation of another, or not. In fact, among possible differentiating factors, one should discriminate between trivalent’s interactions, cosegregation, and cell selection by apoptosis (Anton et al. 2010; Eaker et al. 2001; Merico et al. 2008; Scascitelli et al. 2003). The total frequency of aneuploid spermatids can be determined only by direct experimental analysis with probes for all the involved chromosomes, a task that exceeded the purposes of this article.

Chromosome segregation studies on heterozygote mice are scarce and have been mainly conducted in spermatocytes in metaphase II. Studies with heterozygotes for one, two, three, or four Rb metacentric chromosomes showed that segregational anomalies reach values close to 50 % in metaphase II spermatocytes (Winking et al. 2000; Castiglia and Capanna 2000; Rizzoni and Spirito 1997; Scascitelli et al. 2004). Also, alterations in the alignment during metaphase I have been reported in trivalents from heterozygotes for four Rb translocations (Eaker et al. 2001). Studies on the germinal epithelium of Rb heterozygotes 2n = 31 showed that a ratio of 1 to 4 between the number of spermatocytes and spermatids is lost due to a significant reduction in the number of spermatids (Garagna et al. 2001b). We estimated that in the Rb heterozygote 2n = 32, the number of spermatids is reduced by about 66 % compared with *Mus* 2n = 40 (not shown). Similarly, increased apoptosis of metaphasic spermatocytes in stage XII of the seminiferous epithelium of Rb heterozygotes has been described (Merico et al. 2008). Thus, metaphasic spermatocytes with misaligned chromosomes should account for a significant fraction of apoptotic spermatocytes suggesting that a checkpoint process identifies aberrant meiosis (Eaker et al. 2001). These data together suggest that in multiple Rb heterozygotes, errors in the alignment of chromosomes during metaphases precipitate a large and selective loss of germ cells. Thus, the observed spermatids are those that survived the removal of altered cells; therefore, caution should be exercised in inferring original chromosome segregations from chromosome present.

**Fig. 6:**
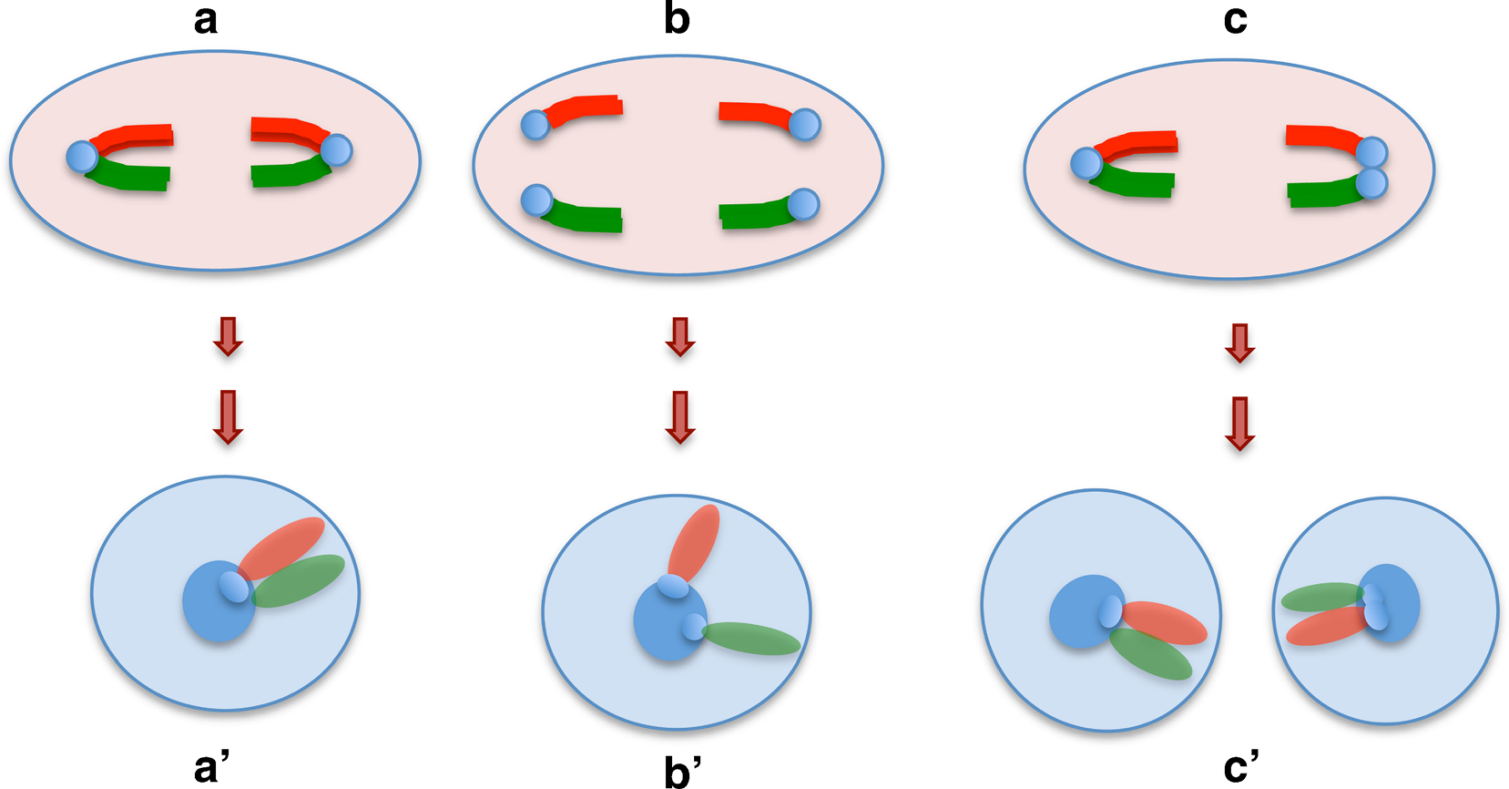
Chromosome arrangement during meiotic segregation and at the spermatid nuclei. The first meiotic anaphase in a standard spermatid nucleus with the expected arrangement of two homologous Rb metacentric chromosomes (*a*); two pairs of homologous telocentric chromosomes (*b*), and one trivalent (*c*). The chromosomes or chromosomal arms are represented in *green* or *red* and the pericentromeric heterochromatin and the chromocenter in the spermatid nuclei is shown in *blue*. In the spermatid nuclei, both arms of the Rb metacentric chromosomes are expected to lie together (*a′*); two telocentric chromosomes should appear to be joined through their heterochromatin (*b′*) and the arms of the Rb chromosome or their telocentric homologs would lie together (*c′*)

The chromosomal painting method applied in this study showed that in about 95 % of the spermatids from 2n = 24 individuals, Rb metacentric chromosomes exhibited a “v shape” with the centromeric regions forming part of a single large chromocenter residing at the nuclear center. This chromocenter is a condensed structure formed by the association of centromeric heterochromatin from all the chromosomes (Hoyer-Fender et al. 2000; Brinkley et al. 1986). In spermatid nuclei of 2n = 40 individuals, the telocentric chromosomes also exhibited a polarized nuclear distribution with a convergence of centromeric regions toward the chromocenter. However, as telocentric chromosomes are independent entities, in only 30 % of the nuclei the studied chromosomal pairs were adjacent or side by side and, in 70 % the observed chromosomes were only connected to each other by a single large chromocenter at the nuclear center (Fig. 5). In most of the spermatid’s nuclei of 2n = 32 individuals, the studied chromosomal pairs appear to be adjacent or side by side in a similar fashion as the Rb metacentric chromosomes in 2n = 24 nuclei. It is possible that in the heterozygote spermatid nuclei this configuration could represent either the metacentric as well as the telocentric chromosomes. Because of chromosomal segregation between metacentrics and the respective homologous telocentrics, together with Rabl polarization (Rabl 1885; Parada and Misteli 2002), the telocentric chromosomal pairs would remain close together during meiotic anaphases and, consequently, in the spermatid’s nucleus exhibiting a “v” shape similar to that of the metacentric chromosomes (Fig. 6). Notwithstanding above, we cannot exclude the possibility that our observations may also be interpreted as a predominance of metacentric chromosomes in spermatids of heterozygotes. Moreover, studies on the transmission of alleles in heterozygotes indicate that Rb chromosomes were transmitted more frequently in males, whereas in females the transmission of telocentric chromosomes would be favored (Underkoffler et al. 2005). In multiple heterozygote male mice, some evidence has been provided suggesting that there is chromosomal cosegregation (Scascitelli et al. 2003). During cosegregation, all Rb metacentric chromosomes are arranged towards the same pole of the cell migrating in the same direction while telocentric chromosomes migrate to the opposite pole (Scascitelli et al. 2003). This has been explained by suggesting that during anaphase I the meiotic spindle is asymmetric, meaning that one of the poles of the cell has a greatest quantity of microtubules, as such, the microtubule-enriched pole will be more efficient anchoring telocentric chromosomes (Scascitelli et al. 2003). In contrast, another study still in progress in our laboratory indicates that segregation of metacentric chromosomes in multiple heterozygotes instead may be random (data not included). Hence, karyotype analyses of 25 descendants from seven crosses between 2n = 32 heterozygotes and 2n = 24 homozygotes already exhibited a wide range of diploid numbers in which metacentrics inherited from the heterozygote’s parent varied from zero to eight and their frequency resembled a normal distribution. If there is no bias during the selection of fertilizing gametes, this would imply that the orientation of metacentric and telocentric in metaphase I for the eight trivalents did not favor one or the other, and that there was no selective advantage for any combination of them.

Considering that the joint expression of ribosomal genes can induce adjacent segregation between nucleolar chromosomes (Mirre et al. 1980; Berríos and Fernández-Donoso 1990), one criterion for the selection of the chromosomes to be studied in *M. musculus domesticus* spermatids and it was their carrying NOR. However, when comparing the frequency of aneuploid spermatids due to chromosomes with or without NOR, no significant differences were found between these two groups. In the trivalent 9 (9.14) 14, none of the chromosomes are carrying NOR; in the trivalent 16 (16.17) 17 and in the trivalent 5 (5.15) 15, only one telocentric and the metacentric arm counterpart to this and carry NOR. During prophase, NORs of a trivalent could participate in the formation of a common nucleolus implying that rDNA could unfold due to active transcription of rDNA leading to intermingling (Berrios et al. 2004). As such, the resulting intertwined chromatin may actually favor the joint segregation of the metacentric and telocentric chromosomes. We stress however that a higher frequency of aneuploidy for these chromosomes was not be observed. Thus in principle, it should be noted that meiotic cells with intermingled nucleolar chromosomes might be eliminated by apoptosis. Moreover, according to our results, the relatively low frequency of gamete aneuploidy observed in mice carriers of multiple Rb chromosomes suggests that a checkpoint mechanism does indeed efficiently eliminated germ cells with chromosomal abnormalities. On the other hand, there are apparently no significant differences in spermatid aneuploidy derived from chromosomes involved in distinct trivalents.

Our data suggest that Rb chromosomes and their telocentric homologs are subjected to architectural constraints forcing them to be close to each other, hence favoring the formation of new Rb metacentric chromosomes. Thus, these observations lend support to the notion of prevalence.
